# Thermodynamically stable amyloid-β monomers have much lower membrane affinity than the small oligomers

**DOI:** 10.3389/fphys.2013.00084

**Published:** 2013-04-18

**Authors:** Bidyut Sarkar, Anand K. Das, Sudipta Maiti

**Affiliations:** Department of Chemical Sciences, Tata Institute of Fundamental ResearchColaba, Mumbai, India

**Keywords:** amyloid beta, peptide oligomerization, peptide-membrane interaction, confocal microscopy, toxicity

## Abstract

Amyloid beta (Aβ) is an extracellular 39–43 residue long peptide present in the mammalian cerebrospinal fluid, whose aggregation is associated with Alzheimer's disease (AD). Small oligomers of Aβ are currently thought to be the key to toxicity. However, it is not clear why the monomers of Aβ are non-toxic, and at what stage of aggregation toxicity emerges. Interactions of Aβ with cell membranes is thought to be the initiator of toxicity, but membrane binding studies with different preparations of monomers and oligomers have not settled this issue. We have earlier found that thermodynamically stable Aβ monomers emerge spontaneously from oligomeric mixtures upon long term incubation in physiological solutions (Nag et al., 2011). Here we show that the membrane-affinity of these stable Aβ monomers is much lower than that of a mixture of monomers and small oligomers (containing dimers to decamers), providing a clue to the emergence of toxicity. Fluorescently labeled Aβ_40_ monomers show negligible binding to cell membranes of a neuronal cell line (RN46A) at physiological concentrations (250 nM), while oligomers at the same concentrations show strong binding within 30 min of incubation. The increased affinity most likely does not require any specific neuronal receptor, since this difference in membrane-affinity was also observed in a somatic cell-line (HEK 293T). Similar results are also obtained for Aβ_42_ monomers and oligomers. Minimal amount of cell death is observed at these concentrations even after 36 h of incubation. It is likely that membrane binding precedes subsequent slower toxic events induced by Aβ. Our results (a) provide an explanation for the non-toxic nature of Aβ monomers, (b) suggest that Aβ toxicity emerges at the initial oligomeric phase, and (c) provide a quick assay for monitoring the benign-to-toxic transformation of Aβ.

## Introduction

Amyloid beta (Aβ), a 39–43 residue long peptide, has been implicated in the etiology of Alzheimer's disease (AD), a progressive neurodegenerative disorder. AD is characterized by the presence of senile plaques (extracellular fibrillar deposits), primarily composed of aggregates of Aβ 1–40 and 1–42 peptides (herein called Aβ_40_ and Aβ_42_, respectively), and intracellular neurofibrillary tangles composed of hyperphosphorylated tau proteins (Selkoe, 1991). Precise identification of the toxic aggregates remains a challenge. Recent studies suggest that the soluble oligomeric species are the key to toxicity (McLean et al., 1999; Lashuel et al., 2002; Rosenblum, 2002; Walsh et al., 2002; Hoshi et al., 2003; Shankar et al., 2007, 2008; Walsh and Selkoe, 2007; Kayed et al., 2009). However, the exact identity of the toxic soluble aggregate species and the mechanism behind the emergence of toxicity are still not clear, as has been articulated in a recent review (Benilova et al., 2012).

We know that the Aβ monomer is present in healthy individuals, and therefore it is unlikely to be toxic. There is some evidence that the monomer is a mixture of random coil and alpha helical parts under non-physiological conditions (Barrow and Zagorski, 1991; Sticht et al., 1995; Coles et al., 1998; Shao et al., 1999; Zhang et al., 2000; Crescenzi et al., 2002; Baumketner et al., 2006; Ball et al., 2011; Vivekanandan et al., 2011; Gu et al., 2012), and some simulation studies (Massi et al., 2001; Straub et al., 2002; Raffa and Rauk, 2007) also provide a similar picture. However, these do not explain why the monomer is non-toxic. The other major question that remains unanswered is at what stage of aggregation the peptide acquires its toxic properties. Different laboratories have identified different types and sizes of toxic oligomers of Aβ, ranging from dimers to protofibrils (McLean et al., 1999; Lashuel et al., 2002; Rosenblum, 2002; Walsh et al., 2002; Hoshi et al., 2003; Shankar et al., 2007, 2008; Walsh and Selkoe, 2007; Kayed et al., 2009). However, these specimens are prepared following different protocols and the toxicity is typically tested with concentrations that are much higher than what is believed to exist *in vivo* (<<1μM). While cell death is a direct and quantitative method to assay toxicity, it is also the last event in the toxic pathway. Some researchers have used Long Term Potentiation (LTP) of neuronal synapses as an earlier functional assay (Walsh et al., 2002; Hung et al., 2008; Shankar et al., 2008). The initial step of Aβ induced toxicity possibly lies in the disruption of the permeability of the cell membrane to specific ions. It has been suggested that this disruption is due to the formation of specific ion channel-like structures in the membrane (Arispe et al., 1993; Kawahara et al., 1997; Sanderson et al., 1997; Bhatia et al., 2000; Quist et al., 2005; Lal et al., 2007; Demuro et al., 2011), though some other studies have suggested that the disruption is caused by a more generic disruption of the membrane architecture (McLaurin and Chakrabartty, 1996; Hertel et al., 1997; Mason et al., 1999; Yip and McLaurin, 2001; Sokolov et al., 2006; Widenbrant et al., 2006; Williams et al., 2011). In any case, since Aβ is an extracellular peptide, measurement of membrane affinity may provide a very early assay, and can potentially identify the benign-to-toxic transition during Aβ aggregation.

Several groups have studied the membrane-affinity of different Aβ aggregate species, but no clear difference between the oligomers and monomers has emerged (Bateman and Chakrabartty, 2009; Nag et al., 2010; Johnson et al., 2011). A recent report suggests that oligomers are more likely to attach to the membrane than the monomers (Narayan et al., 2013). However, this experiment also used a mixture of monomers and oligomers. It is possible that a more stringent verification of the monomeric nature of the peptide solution, and/or adequate equilibration of this species in a low concentration physiological buffer may bring out the differences better, and give quantitative results. We have earlier found that oligomers spontaneously dissociates into stable monomeric species when they are incubated at a low concentration for a long time (>1 week) in a physiological buffer solution (Nag et al., 2011). It is thus possible to make nearly pure monomeric solutions, where the monomer conformation is in a stable equilibrium. This stable monomeric species is likely to be similar to what exists *in vivo*. Here we compare its membrane-affinity with the oligomeric species.

We characterize the monomers and the small oligomers of fluorescently labeled Aβ_40_ peptides by Fluorescence Correlation Spectroscopy (FCS), as described earlier (Nag et al., 2011). We subsequently measure the relative affinity of the two distinct aggregation states to live cell membranes using confocal imaging at near-physiological sub-μM concentrations. We also assay the toxicity of these species to the serotonergic neuron-derived cell line RN46A. We find that while there is minimal cell death at these concentrations, the monomeric species exhibits at least 25× lower affinity for the cell membrane. The experiments are then repeated for a somatic cell line (HEK293T), which also yield similar results. We further repeat the experiments with the Aβ_42_ species, and obtain very similar results. We infer that membrane affinity is a key functional difference between the benign monomers and the cytotoxic aggregates. The smallest group of oligomers that we can clearly identify in our experiment (a mixture dominated by tetramers) has already undergone this benign-to-toxic transformation.

## Materials and methods

### Materials

9-fluorenylmethoxycarbonyl (Fmoc) protected amino acids, rink amide MBHA resin LL (0.31 mmol/g), 2-(7-Aza-1H-benzotriazole-1-yl)-1,1,3,3-tetramethyluroniumhexafluorophosphate (HATU), O-benzotriazole-N,N,N′,N′-tetramethyluroniumhexafluorophosphate (HBTU), and triisopropylsilane (TIS) were purchased from Merck (Schuchardt OHG, Germany). N,N-dimethylformamide (DMF), N-methyl morpholine (NMM), piperidine, trifluoroacetic acid (TFA), tert-butyl methyl ether, acetonitrile, isopropyl alcohol (IPA), disodium hydrogen orthophosphate dihydrate (Na_2_HPO_4_, 2H_2_O), and potassium dihydrogen orthophosphate (KH_2_PO_4_) were obtained from S.D. Fine Chem. Ltd. (Mumbai, India). Sodium chloride (NaCl) is obtained from Fisher Scientific (Mumbai, India). Potassium Chloride (KCl) and calcium chloride dihydrate (CaCl_2_, 2H_2_O) were purchased from SRL (Mumbai, India). Magnesium sulphate (MgSO_4_, 7H_2_O) was obtained from AnalaR, Glaxo laboratories (Mumbai, India). 1,8-diazabicyclo[5.4.0]undec-7-ene (DBU), hexafluoroisopropanol (HFIP), 4-(2-hydroxyethyl)-1-piperazineethanesulfonic acid (HEPES), cholesterol, Nile red, propidium iodide, dextrose, poly-L-Lysine, and Dulbecco's Modified Eagle Medium (DMEM) were purchased from Sigma-Aldrich Inc. (St. Louis, MO, USA). Phenol, ethane dithiol (EDT), thioanisole and trifluoroethanol (TFE) were purchased from Fluka (St. Louis, MO, USA). Hoechst 33342 was purchased from Molecular Probes (OR, USA). PenStrep, Fetal Bovine Serum (FBS), DMEM-F12 media, and Trypsin were purchased from Gibco (Grand Island, NY, USA). Rhodamine labeled Aβ_1−40_ (R-Aβ_40_) and Aβ_1−42_ (R-Aβ_42_), Fluorescein labeled Aβ_1−40_ (F-Aβ_40_) were purchased from rPeptide Inc. (San Jose, CA, USA).

### Peptide synthesis and purification

Aβ_40_ was synthesized using standard Fmoc chemistry in an automated solid phase peptide synthesizer (PS3, Protein technologies Inc., USA), as described previously (Mithu et al., 2011). Briefly, Rink amide MBHA resin LL (0.31 mmol/g) was used as solid support to grow the peptide chains. 4-fold excess Fmoc amino acids were activated with equimolar HATU or HBTU and NMM (0.4 M) in DMF. Fmoc deprotection was carried out using mixture of 2% DBU and 20% piperidine in DMF (v/v). A mixture containing TFA, TIS, water, EDT, thioanisole and phenol at a volume ratio of 32:1:2:1:2:2 was used for the cleavage of the peptide from the resin and deprotection of the acid labile side chains (mixing time = 4 h). The peptide was concentrated under nitrogen flow, and precipitated and washed with tert-butyl methyl ether. The precipitates were dried under vacuum to obtain powdered crude peptides. The crude peptide was dissolved in a HFIP/TFE mixture (1:3, v/v) and purified by reverse phase high performance liquid chromatography (Prominence 20A, Shimadzu Corporation, Kyoto, Japan). Separation was achieved by passing the crude peptide through a C4 semi-preparative column (Kromasil, Eka Chemicals AB, Bohus, Sweden) and using a gradient of acetonitrile and 0.1% TFA in water as the eluent. The purity of the peptide was verified by matrix assisted laser desorption ionization-time of flight MS (Model: TOF SPEC 2E, Micromass, Manchester, England).

### Preparation of specimens with different sizes

**Figure A1:**
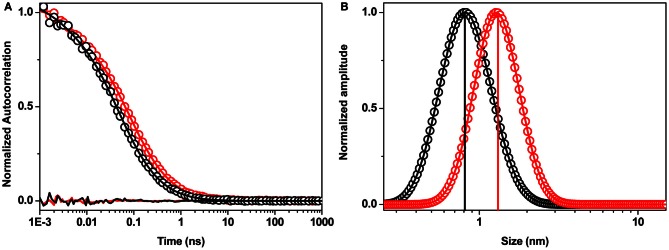
**Size of Aβ aggregates. (A)** Fluorescence correlation spectroscopy data (circles) of Aβ_40_ monomers (190 days old, black) and small oligomers (30 min old, red). MEMFCS fit (solid line) and residuals (below the curve) are shown in corresponding colors. **(B)** Size distribution of monomers (black) and small oligomers (red) obtained from the MEMFCS fit in **(A)**. The sizes determined from a single component fit to the FCS data are shown using corresponding colored bars.

The preparation and characterization of the monomeric and the small oligomeric species have been described elsewhere (Nag et al., 2011). We note that both were prepared in aqueous buffer (modified Thomson's buffer (TB) consisting of 20 mM sodium HEPES, 146 mM NaCl, 5.4 mM KCl, 1.8 mM CaCl_2_, 0.8 mM MgSO_4_, 0.4 mM KH_2_PO_4_, 0.3 mM Na_2_HPO_4_, and 5 mM dextrose; pH adjusted to 7.4). They exist in a quasi-equilibrium state, and no size based separation was carried out. Briefly, fluorophore labeled peptides were initially dissolved in water at pH 11 (adjusted by NaOH) to prepare stock solutions of 100 μM and diluted to 250 (or 50) nM in physiological buffer solutions at pH 7.4 at 25°C. The time of this dilution (and consequent pH change to ~7.4) was taken as the initial time point of the measurement. The sizes were measured by FCS of rhodamine (or fluorescein) labeled Aβ. We had earlier shown before that at sub-saturated concentrations the initial particles are small oligomers (2–10 mers) and they spontaneously dissociate to monomers over ~5 days (Nag et al., 2011). Initially, the average particle size of R-Aβ_40_ was about 1.35 ± 0.20 nm (Figure A1, red), which evolved over several days to reach a steady particle size of about 0.8 nm. No further change of this size was observed in more than six months (190 days) of incubation (Figure A1, black). Observations with R-Aβ_42_ were similar. The membrane binding experiments were performed with pure R-Aβ_40/42_ at 250 nM of monomers (>4-weeks old) and small oligomers. Toxicity experiments were performed on RN46A cells with 250 nM and 100 μM of Aβ_40_.

### FCS measurements

FCS Measurements were performed with an instrument constructed in-house (Sengupta et al., 2002). Briefly, a 543 nm laser (He-Ne) beam was expanded and collimated before focusing into the sample volume using an apochromatic 60 × water immersion objective with numerical aperture of 1.2 (Olympus, PA, USA). The fluorescence was collected using the same objective, separated from the excitation beam by a dichroic mirror (Chroma, VT, USA) and focused onto a 25 μm core-diameter optical fiber after filtered using suitable emission filter (Chroma, VT, USA). The fiber was used as a confocal pinhole to reject the out of focus fluorescence. The fluorescence was detected by a single photon avalanche photodiode (APD, PerkinElmer Inc., Waltham, MA, USA) and the data were collected and processed using a hardware correlator card (ALV 5000E, ALV Laser, VmbH, Langen, Germany). FCS data were fitted either with a discrete few-component diffusion model in Origin 7.5 software (OriginLab, Northampton, MA, USA), and/or with a Maximum Entropy Method (MEM) based fitting routine written in house (Sengupta et al., 2003) to obtain diffusion times. The diffusion times were converted into hydrodynamic radii (*R*_*h*_) using rhodamine B [*R*_*h*_ = 0.57 nm (Culbertson et al., 2002)] as a calibrant. Sizes of F-Aβ_40_ species were determined similarly from a set up constructed using Ar-ion laser (488 nm) and appropriate dichroic mirror and filter sets.

### Cell culture

Human Embryonic Kidney 293T (HEK293T) cells were cultured in DMEM supplemented with 10% FBS, 50 units/ml Penicillin and 50 μg/ml Streptomycin at 37°C under humidified air containing 5% CO_2_ in T-25 canted-neck flasks. For RN46A cells DMEM-F12 (1:1) was used instead of regular DMEM media. For membrane binding studies both types of cells were cultured in home-made cover slip-bottomed Petri dish coated with poly-L-Lysine (0.1 mg/ml). Cells for toxicity measurements were grown in 96 well plates.

### Membrane affinity studies

HEK293T and RN46A cells were imaged in a confocal microscope (LSM-710, Carl Zeiss, Germany) using 63× oil immersion or 40× water immersion objectives. 543 nm light was used to excite R-Aβ_40/42_ and the fluorescence was collected between 550 and 700 nm. Fluorescence images were recorded at an interval of 1 μm (or 0.5 μm) in the longitudinal (Z) direction. The cells were initially imaged in TB (zero time data). Subsequently, after 30 min of incubation with 250 nM monomeric or oligomeric R-Aβ_40/42_ solution, the dishes were washed with TB and imaged again. Control dishes were sham-treated with TB. The brightness of the membrane region of a cell (at the brightest Z-position) was analyzed after subtraction of the non-cell background, using ImageJ (open source software, available from the website http://rsbweb.nih.gov/ij/). For the 30 h membrane binding experiments, 1 μM solutions of monomeric (or oligomeric) R-Aβ_40_ were diluted to 50 nM in the cell culture media. After 30 h of incubation the cells were washed with TB and imaged as described. Comparison of the membrane brightness yielded the relative binding of monomers and oligomers. We report the analysis of 18 or more cells from ≥3 independent measurements for each experiment.

For membrane co-localization studies, RN46A cells were incubated with 50 nM oligomeric F-Aβ_40_ for 30 min, washed with TB and imaged for fluorescein (excitation 488 nm, emission 496–535 nm). Nile red (a membrane labeling dye, 300 nM) was added to the cells and imaged again after 5 min (excitation 543 nm, emission 650–720 nm). Leak of fluorescein fluorescence into the Nile red channel by direct excitation with 543 nm was checked prior to Nile red addition, and was found to be negligible.

### Toxicity measurements

RN46A cells were treated with freshly prepared solutions of unlabeled Aβ_40_ at 250 nM and 100 μM concentrations in cell culture media. The cells were assessed after 36 h for the extent of cell death. The cells were treated with 0.01 mg/ml concentrations of Hoechst 33342 (a DNA intercalating dye that permeates membranes and hence label all the cells present) and propidium iodide (PI, another DNA intercalating dye that does not permeate live membranes and hence labels only the dead cells with damaged membranes) in TB for 10 min followed by washing with TB. The cells were fixed at this stage using 4% para-formaldehyde (PFA) using standard procedure. The cells were imaged for Hoechst 33342 and PI fluorescence in a confocal microscope setup (LSM-710, Zeiss, Germany) using a 20× objective. 690 nm pulsed light form a mode-locked Ti-sapphire laser (MaiTai, Spectra Physics, CA, USA) was used for the two-photon excitation of Hoechst 33342. The fluorescence was separated from the excitation using a 690 nm+ dichroic mirror and detected using a photomultiplier tube (385–535 nm). PI was excited using a 543 nm laser (He-Ne, Zeiss), the fluorescence was separated using a dichroic mirror and detected between 565 and 720 nm. Images were analyzed for the total number of cells (Hoechst 33342 fluorescent spots) and the number dead cells (PI fluorescent spots) using an automated particle counter in ImageJ. The ratio of cells which are alive (PI negative) to total cells was reported as the % viability. The analysis was performed on a total of 3510, 4720, 4795 number of cells for sham, 250 nM Aβ_40_ and 100 μM Aβ_40_ treatments, respectively.

## Results

### Membrane affinity measurement

**Figure 1:**
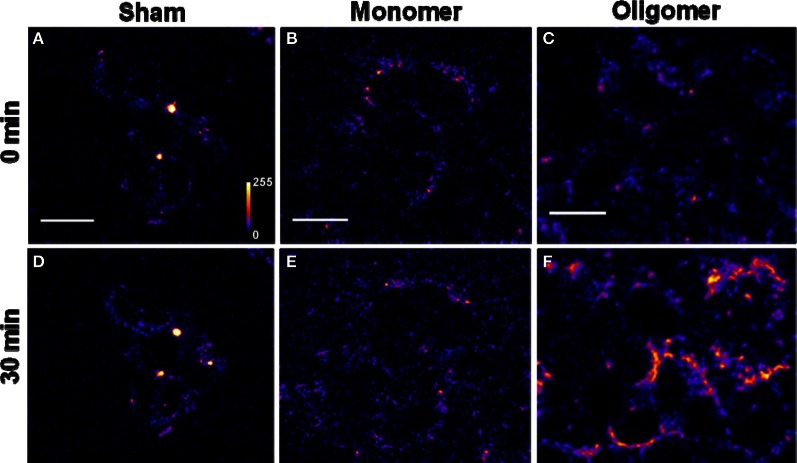
**Binding of Aβ_40_ monomers and oligomers to HEK293T cell membranes. (A–C)** Confocal sections of three sets of cells (excitation 543 nm, emission 550–700 nm). **(D–F)** Corresponding sets of cells after 30 min of incubation with buffer **(D)**, 250 nM of monomeric R-Aβ_40_ **(E)**, and 250 nM of small oligomeric R-Aβ_40_ **(F)**. Intensity is false color coded. Scale bar = 20 μm.

**Figure 2:**
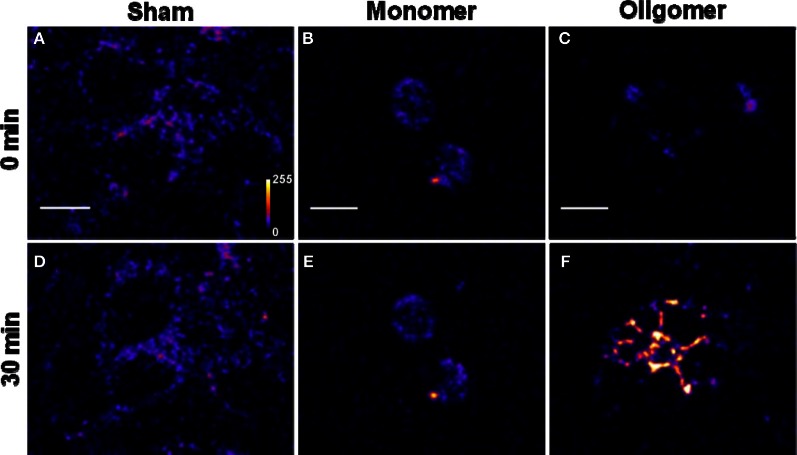
**Binding of Aβ_42_ monomers and oligomers to HEK293T cell membranes. (A–C)** Confocal sections of three sets of cells (excitation 543 nm, emission 550–700 nm). **(D–F)** Corresponding sets of cells after 30 min of incubation with buffer **(D)**, 250 nM of monomeric R-Aβ_42_ **(E)**, and 250 nM of small oligomeric R-Aβ_42_ **(F)**. Intensity is false color coded. Scale bar = 10 μm.

**Figure 3:**
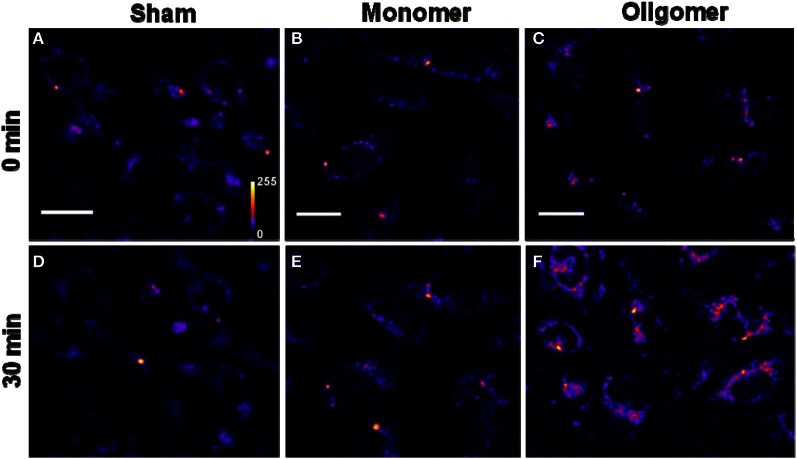
**Binding of Aβ_40_ monomers and oligomers to RN46A cell membranes. (A–C)** Confocal sections of three sets of cells (excitation 543 nm, emission 550–700 nm). **(D–F)** Corresponding sets of cells after 30 min of incubation with buffer **(D)**, 250 nM of monomeric R-Aβ_40_ **(E)**, and 250 nM of small oligomeric R-Aβ_40_ **(F)**. Intensity is false color coded. Scale bar = 20 μm.

**Figure 4:**
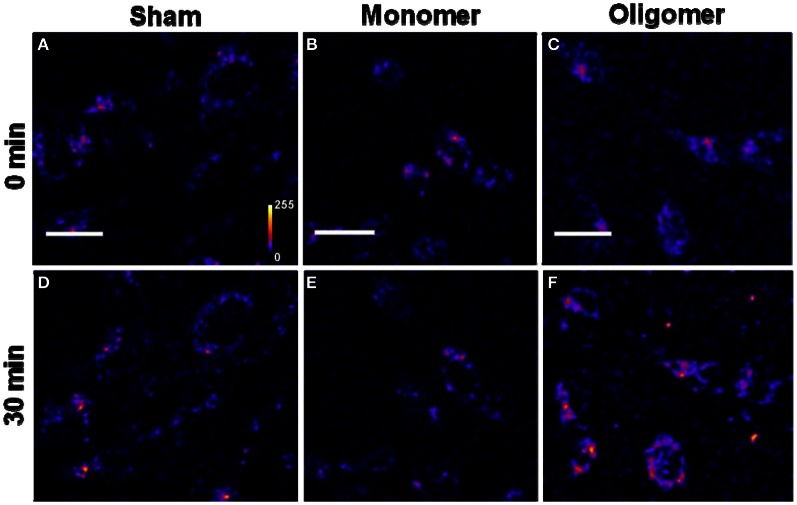
**Binding of Aβ_42_ monomers and oligomers to RN46A cell membranes. (A–C)** Confocal sections of three sets of cells (excitation 543 nm, emission 550–700 nm). **(D–F)** Corresponding sets of cells after 30 min of incubation with buffer **(D)**, 250 nM of monomeric R-Aβ_42_ **(E)**, and 250 nM of small oligomeric R-Aβ_42_ **(F)**. Intensity is false color coded. Scale bar = 20 μm.

**Figure 5:**
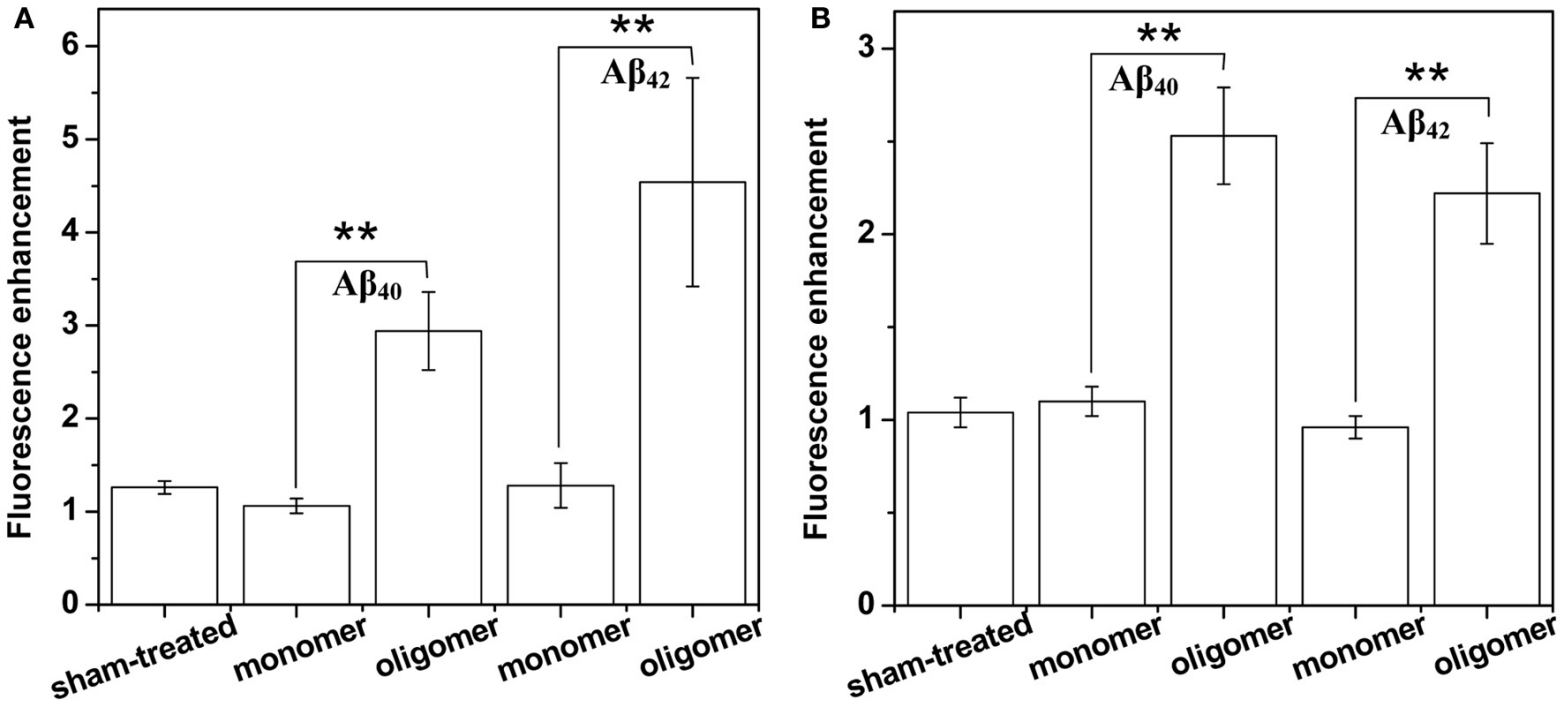
**Summary of membrane affinity measurements of Aβ monomers and oligomers.** Increase of membrane brightness of **(A)** HEK293 cells and **(B)** RN46A cells after 30 min exposure to buffer (first bar), 250 nM monomeric and oligomeric RAβ_40_ (second and third bars, respectively), and 250 nM monomeric and oligomeric RAβ_42_ (fourth and fifth bars, respectively). Values represent mean ± sem. ^**^Indicates that the differences are statistically significant (*P* ≤ 0.01).

**Figure A2:**
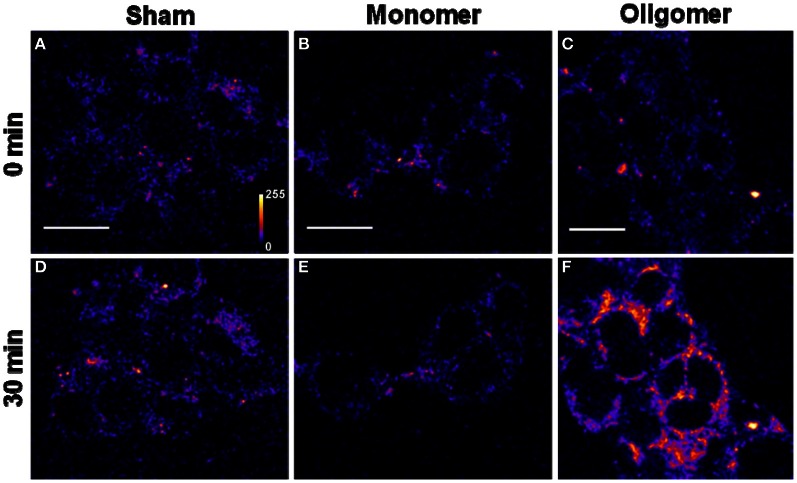
**Binding of Aβ_40_ monomers and oligomers to HEK293T cell membranes. (A–C)** Confocal sections of three sets of cells (excitation 543 nm, emission 550–700 nm). **(D–F)** Corresponding sets of cells after 30 min of incubation with buffer **(D), 50 nM of monomeric** R-Aβ_40_ **(E)**, and 50 nM of small oligomeric R-Aβ_40_ **(F)**. Intensity is false color coded. Scale bar = 20 μm.

**Figure A3:**
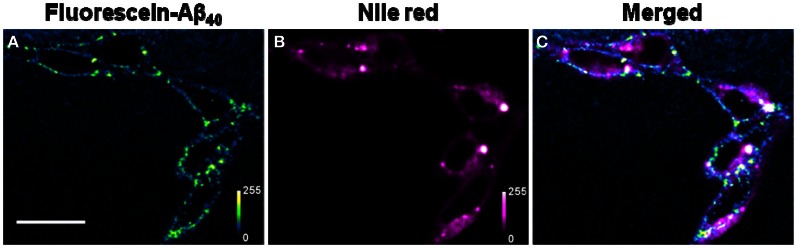
**Location of Aβ aggregates.** Confocal image of **(A)** RN46A cells treated with 50 nM of oligomeric F-Aβ_40_ for 30 min, **(B)** same cells incubated with 300 nM Nile red following F-Aβ_40_ treatment in **(A)**. **(C)** Co-localization of F-Aβ_40_ (blue-green false color coded) and Nile red (magenta false color coded). Scale bar = 20 μm.

- *Binding of Aβ_40_ to cultured HEK293T cells:* HEK293T (a cell line of somatic origin) cells were incubated with modified TB containing 250 nM R-Aβ_40_ monomers (or small oligomers) at room temperature for 30 min. Figure 1 shows the membrane regions of the cells imaged with a confocal microscope, both at the initial time (monomers, Figure 1B, oligomers, Figure 1C) and after 30 min of incubation (monomers, Figure 1E, oligomers, Figure 1F). Figures 1A and 1D show a sham treated control set of cells at 0 and 30 min, respectively. The fluorescence intensity is false-color coded in all the images. The degree of binding of the peptide to the cell membrane was assessed by analyzing the change in the brightness of the membrane during the incubation period. We observed that the membranes of the cells incubated with the oligomers brighten up considerably (by a factor of 2.94 ± 0.42, Figure 1C vs. Figure 1F). On the other hand, the cells incubated with monomers showed a negligible change in brightness (factor of 1.06 ± 0.08, Figure 1B vs. Figure 1E). This is similar to the sham-treated cells (factor of 1.26 ± 0.07, Figure 1A vs. Figure 1D). The results are summarized in a bar graph (Figure 5A). The first three bars show the fluorescence enhancement after 30 min (relative to 0 min) for treatment with sham, 250 nM Aβ_40_ monomers and 250 nM Aβ_40_ oligomers respectively.
  Qualitatively similar results were obtained even at a lower peptide concentration of 50 nM (Figure A2), as well as for a longer incubation time of 30 h (data not shown).
- *Binding of Aβ_42_ to cultured HEK293T cells:* Similar experiments were repeated for 250 nM R-Aβ_42_ (Figure 2), which is the more aggregation-prone variant of Aβ, and is the dominant species present in the cerebral plaques of Alzheimer's patients (Burdick et al., 1992; Jarrett et al., 1993). Figures 2A, 2B, and 2C show cells treated with sham, monomers, and oligomers, respectively, at zero-time. Figures 2D, 2E, and 2F show the same cells after 30 min. The membranes of the cells incubated with the oligomers brighten up by approximately a factor of 4.5 ± 1.1 (Figure 2C vs. Figure 2F). The cells incubated with monomers showed a negligible change in their brightness (factor of 1.28 ± 0.24, Figure 2B vs. Figure 2E). The results are summarized in Figure 5A, where the last two bars correspond to the treatment with Aβ_42_ monomers and oligomers, respectively.
- *Binding of Aβ_40_ to cultured RN46A cells:* We then asked if the monomers would bind better to a cell line of neuronal origin, namely RN46A. These cells are derived from the serotonergic neurons of the rat (White et al., 1994), and have been characterized for their neurotransmitter content and activity by us earlier (Balaji et al., 2005). Figures 3A, 3B, and 3C show cells treated with sham, monomers (250 nM), and oligomers (250 nM), respectively, at zero-time. Figures 3D, 3E, and 3F show the same cells after 30 min. Membranes of the cells incubated with the oligomers brighten up by approximately a factor of 2.53 ± 0.26 (Figure 3C vs. Figure 3F). On the other hand, the cells incubated with monomers showed a negligible change in brightness (factor of 1.10 ± 0.08, Figure 3B vs. Figure 3E). This is similar to the sham-treated cells (factor of 1.04 ± 0.08, Figure 3A vs. Figure 3D). The first three bars in Figure 5B show the relative binding of the sham, the monomers and the oligomers, respectively.
- *Binding of Aβ_42_ to cultured RN46A cells:* Figures 4A, 4B, and 4C show cells treated with sham, R-Aβ_42_ monomers (250 nM), and oligomers (250 nM), respectively, at zero-time. Figures 4D, 4E, and 4F show the same cells after 30 min. The membranes of the cells incubated with the oligomers brighten up by approximately a factor of 2.22 ± 0.27 (Figure 4C vs. Figure 4F). The cells incubated with monomers showed a negligible change in their brightness (factor of 0.96 ± 0.06, Figure 4B vs. Figure 4E). The last two bars in Figure 5B correspond to treatment with Aβ_42_ monomers and oligomers, respectively.
- *Location of Aβ on the cells:* The ring shaped brightness observed in the confocal slices suggest that the Aβ is primarily localized on the membrane (Figure A3). For further verification, we incubate the cells with 300 nM of the membrane labelling dye Nile Red (Figure A3B) for 5 min, subsequent to their exposure to 50 nM of F-Aβ_40_ oligomers (Figure A3A). The sequential imaging for F-Aβ_40_ and Nile red suggests that Aβ primarily localizes on the membrane. Though the Aβ_40_ staining appears more punctate, bright spots (false color coded) in Figure A3C show considerable co-localization.

### Toxicity measurements

Toxicity measurements were carried out for 250 nM and 100 μM of oligomeric Aβ_40_ on RN46A cells as described in the methods section. The ratio of the number of cells marked with PI to the cells marked with Hoechst 33342 provides a measurement for the percentage of dead cells. 85.2 ± 3.9% cells remains viable after 250 nM Aβ_40_ treatment for 36 h. This is similar to sham-treatment which shows 88.4 ± 3.2% cell viability, while the toxicity is clearly observed at 100 μM, which shows 27.6 ± 10.0% viability.

## Discussion

We had earlier established that a mixture of monomers and small oligomers of Aβ_40_ can attach to cell membranes at low physiological concentrations (Nag et al., 2010). We are now able to separate the contribution of the monomers. Current results establish that the oligomers bind with much higher affinity than the monomers at physiological concentrations (~250 nM and even at 50 nM) (Figures 1, 3, and A2). The monomers do not bind even after longer incubation (30 h, data not shown). Aβ_42_ monomers also show a similarly low membrane-affinity compared to the oligomers (Figures 2 and 4). Figure 5 summarize the relative affinity of different species to cell membranes. The oligomeric solution is a mixture of small n-mers (2 < *n* < 10), with the distribution peaking around the tetramer (Figure A1B, red). Therefore this transformation of the membrane-affinity happens fairly early in the aggregation process. Similar results are also obtained with Aβ_42_, pointing toward a common step in the evolution of toxicity in AD.

There have been conflicting reports in the literature about the comparative ability of the monomers vs. the oligomers to bind lipid membranes. It has been suggested that Aβ oligomers can make cell membranes permeable to Ca^++^ ions, while monomers cannot (Demuro et al., 2011). However, Yip and coworkers suggested that Aβ_40_ monomers incorporate into artificial membranes and disrupt it (Yip and McLaurin, 2001). Recent studies by Gafni, Steel and coworkers, who use elegant single molecule brightness analysis, show that a monomer-rich species can bind to black lipid membranes (Schauerte et al., 2010), and also to live cells (Johnson et al., 2011). In the latter study, the cell-membrane bound species ranged from monomers to hexamers and larger, with dimers to tetramers being the dominant species. In a recent study using brightness and two-color coincidence analysis, Narayan and coworkers have suggested that oligomers preferentially bind to the cell membranes (Narayan et al., 2013). However, no “monomer only” solution was studied in these experiments, and the monomers may not be the same as the long-time equilibrated species we report here. Also, Zhang et al. (2012) have suggested that it is the monomers and not the oligomers that bind to the cell membranes. The reported results are therefore somewhat contradictory. In addition, these studies do not quantify the size of the monomers and oligomers in solution phase. In some of these studies, what is reported as monomers may have had an admixture of small oligomers. For example, two-color coincidence detection would not distinguish between a monomer and a homo-oligomer. It is also important to note that most of the protocols use size-exclusion based separations of monomers and oligomers, which take the system away from the equilibrium between inter-converting species. It is possible that this equilibrium is partially re-established during actual membrane affinity measurements (therefore re-introducing oligomers in the putative monomeric solution) after such a separation step. This is more likely to happen at high (≥ μM) concentrations used in most of these experiments. In many cases, the monomer size is not quantitatively characterized to establish its monomeric nature, and its conformation may not have reached equilibrium. We have earlier found that a nearly pure monomeric solution can be formed by allowing an oligomeric species to spontaneously dissociate at low concentrations for many days (Nag et al., 2011). In our experiments, the separation is spontaneous, as the monomers evolve slowly (over days) from the oligomeric solution. This size subsequently does not change at least up to 6 months (Figure A1, black). This shows that these monomers are a truly thermodynamically stable species. The oligomers are also equilibrated in the buffer for 30–60 min before any experiment is carried out. Of course the oligomeric solution is actually a mixture of monomers and oligomers, but given the size distribution (Figure A1, red), it appears that a species with about 1.35 nm size dominates (this corresponds to a tetramer, if we assume an approximate spherical shape).

It is interesting to speculate why the monomers have so much lower membrane-affinity compared to the oligomers. It is likely that monomers and oligomers differ not just in size, but also in conformation, and the conformational aspects may be the key to understanding the increased membrane-affinity for the oligomers. However, there is no conclusive measurement of the structure of the monomers and the oligomers under physiological conditions. Hard and coworkers have suggested that forcing the monomer into a hairpin-like structure increases its toxicity (Sandberg et al., 2010). Smith and coworkers have suggested that different oligomeric species of similar size can have dissimilar toxicity (Ladiwala et al., 2012), pointing toward the role played by folding. They have also proposed a specific pentameric oligomer model in which the monomers have a conformation which is different than that in the fibrils (Ahmed et al., 2010). Recent work in our laboratory suggests that the inter-terminal distance of Aβ is drastically different between the monomers and the oligomers (Nag, unpublished), and such conformational change may be the key to the increased affinity.

It has been suggested that Aβ binds to specific cell surface receptors (Shankar et al., 2007; Lauren et al., 2009; Decker et al., 2010; Dinamarca et al., 2011; Tong et al., 2011). Interestingly, our studies with a neuronal cell line (RN46A) and a somatic cell line (HEK293T) show very similar results. This suggests that specific neuronal receptors may not be essential for membrane binding. The toxicity studies at physiological solutions do not indicate any significant enhancement in cell-death at these physiological sub-μM concentrations even after at 36 h. This is consistent with observations made by others earlier. Most studies yield considerable cell death only at >1 μM concentrations (Lin et al., 2001). This may seem to be a contradiction, given that the physiological concentrations are usually estimated to be 250 nM or less (Lue et al., 1999; Shankar et al., 2009). However, one has to also keep in mind that the *in vivo* toxicity perhaps takes a much longer time-scale to evolve than is possible to replicate in a laboratory cell culture experiment. At sub-μM concentrations, LTP impairment and neurite disruption provides an assay for Aβ toxicity (Lin et al., 2001; Walsh et al., 2002; Hung et al., 2008; Shankar et al., 2008). However, these measurements require neuronal cultures, and are relatively complex. On the other hand, Aβ is believed to mostly exist in the extracellular space (in the cerebro-spinal fluid), and therefore an interaction with the cell membrane appears to be an obligatory step in toxicity. Given that the non-toxic monomers do not bind, it appears likely that membrane binding signals a very early event in the toxic cascade of Aβ aggregation which ultimately leads to pathogenicity. In that case, membrane binding would provide a simple and quantitative assay for the onset of toxicity.

## Conclusion

We conclude that the Aβ monomer is non-toxic because it has low affinity to cell membranes. The smallest aggregated species that we can distinguish (2–10 mers) are able to attack the cell membrane. Our results suggest that that the monomer to oligomer transition is potentially the most biologically significant step in the aggregation cascade of Aβ. We propose that the enhanced membrane affinity of oligomeric Aβ can be a powerful but simple assay for the emergence of Aβ bioactivity.

### Conflict of interest statement

The authors declare that the research was conducted in the absence of any commercial or financial relationships that could be construed as a potential conflict of interest.
